# Progress in Disease of Digestive Organs

**Published:** 1900-12-01

**Authors:** 


### Progress in Disease of Digestive Organs.

**Mouth and Œsophagus.**—W. Hunter,[1] writing on oral sepsis as a cause of disease, claims that virulent organisms from decayed teeth and dental fittings continuously passing into the stomach lead to various septicæmic states. He asks, Where do the organisms which cause so many of our diseases attain virulence, for they may be found in an attenuated form on the skin, for example, of healthy persons? In dental cavities this may often take place, as they form an ideal culture bed. Aitchison Robertson[2] has estimated the starch-digesting power of the saliva in various diseases. In acid dyspepsia it was in excess, in ulcer of the stomach slightly subnormal, in chronic gastric catarrh variable, in dilatation it was absent. Hepatic cirrhosis showed sometimes an increase; in pulmonary and heart diseases it was fairly normal, with an excess in pneumonia. There was deficient power in nerve and kidney affections, and excess was seen in diabetes. In anæmia with dyspeptic symptoms a deficiency was found; and in fevers, though the quantity of saliva was lessened, its activity was good. F. A. R. Jung[3] shows the great difficulty of diagnosing diverticula of the lower part of the œsophagus. Pressure diverticula usually occur in the upper third, while idiopathic dilatations are rare anywhere. The various tubes which have been devised, gastrodiaphanes, X-rays, and endoscopes may be employed. If food eaten two or three days before is vomited while later meals remain down, there is a diverticulum. Again, too, after emptying the œsophagus a tube will often enter the stomach, and the swallowing sounds are often normal, while in dilatation they disappear. In these affections there may be painful cardiac spasms. V. Blum[4] diagnosed a diverticulum and its size by the X-rays, introducing first a tube of mercury, then a sound filled with bismuth; and, thus directed, he succeeded in passing in a bag filled with a solution of potassium bromide, which is opaque to X-rays, and gave the dimensions of the cavity.

**Cancer.**—H. Strauss[5] lays down that much pus in the stomach contents is usually due to cancer. It is found chiefly in cases where there is no disturbance of motor powers, and these are just the cases where there are few available symptoms. The presence of blood, too, in the fasting stomach may be of value as another sign of cancer. C. Douglas notices that a blood count shows the reduction of red corpuscles and hæmoglobin in this disease, while the leucocytes remain at their normal number or may be increased. There is an early loss of flesh and of appetite, as well as the frequent absence of HCl and the presence of lactic acid. Operative treatment must as a rule be undertaken before there is a palpable tumour. Hence the importance of an early diagnosis, as Schrady remarks.[6] Besides chronic dyspepsia, lactic acid, and reduction o the HCl, we may find irregular mitoses in the epithelial cells after washing out the stomach. If we ask what diseases besides cancer show excess of lactic acid, Paul Cohnheim replies,[7] (1) chronic gastritis with pyloric stenosis, but this is very rare; (2) tumours elsewhere which press on the pylorus, if atrophy of the mucosa is present from the cachexia. But either of these cases will benefit from operation. Recent German statistics of operations on 173 patients show a mortality of only 31 per cent., and several patients remained well for four, five, and eight years. B. F. Curtis[8] relies for diagnosis chiefly on the chemical tests, and in deciding upon operation he would consider the presence of a tumour, obstinate vomiting, especially of blood, and the failure of motor power as important symptoms.

About 2·5 per cent. of all cases of cancer of the stomach seem to occur in persons under thirty, according to Osler,[9] but it is extremely rare under ten. Indeed only six cases are known, while seventeen instances of hypertrophy of the pylorus in infants have been collected, an affection which may simulate malignant disease from its rapid course. There are only 13 cases of cancer recorded in the second decade of life, while in the third they become rather more frequent. On the whole, Osler remarks that the disease in the young often runs an acute and rapid course, and in many there is an abrupt onset. Loss of appetite does not seem a frequent symptom, pain, vomiting, and fever may or may not be present, ascites did not occur in Osler's own cases, and, indeed, the disease is probably often overlooked. Very different is the incidence of cancer during later life, for, next to the uterus, the stomach is the most frequent seat of carcinoma, showing over 21 per cent. in 13,500 cases. Bryant[10] classifies the various forms as (*a*) cancer of the pylorus, (*b*) of the cardiac end, (*c*) local, and (*d*) diffuse. In the first we may have dilatation of the stomach with peristalsis, a vomiting of large amounts of fluid, also frequent and profuse hæmatemesis and possibly a tumour. In (*b*) there is often dysphagia and regurgitation, rarely hæmorrhage or tumour. In local carcinoma the orifices are free, and unless a tumour or hæmorrhage occur there are few symptoms. The diffuse form is marked by a contraction of the stomach, even the abdomen may be retracted, and hœmorrhage is usually absent.

[1] Brit. Med. Jour., July 25. [2] Lancet, May 12. [3] Amer. Jour. Med. Sci., April. [4] Brit. Med. Jour., June 9. [5] Amer. Jour. Med. Sci., May. [6] Med. Rec., Sept 18. [7] New York Med. Jour., June 16. [8] Med. Rec., March 17. [9] New York Med. Jour., April 21. [10] Clin. Jour., March 21.

